# Determinants of retention in care in an antiretroviral therapy (ART) program in urban Cameroon, 2003–2005

**Published:** 2008-07-04

**Authors:** Landry Tsague, Sinata S Koulla, Alain Kenfak, Charles Kouanfack, Mathurin Tejiokem, Therese Abong, Madeleine Mbangue, Yacouba Njankouo Mapoure, Claudine Essomba, Jembia Mosoko, Regis Pouillot, Louis Menyeng, Helene Epee, Carno Tchuani, Anne Cecile Zoung-Kanyi, Lucienne Assumpta Bella, Leopold Zekeng

**Affiliations:** 1 Directorate for Disease Control, Ministry of Public Health, Cameroon;; 2 Faculty of Medicine and Biomedical Sciences, University of Yaoundé I, Cameroon;; 3 Yaounde Central Hospital;; 4 National AIDS Control Program;; 5 Centre Pasteur du Cameroun, Yaoundé;; 6 Douala Laquintinie Hospital;

**Keywords:** HIV, HAART, low income country, retention in care, loss to follow-up, cohort studies, Cameroon, sub-Sahara Africa

## Abstract

**Background:**

Retention in long-term antiretroviral therapy (ART) program remains a major challenge for effective management of HIV infected people in sub-Saharan Africa. Highly Active Antiretroviral Therapy (ART) discontinuation raises concerns about drug resistance and could negate much of the benefit sought by ART programs.

**Methods:**

Based on existing patient records, we assessed determinants of retention in HIV care among HIV patients enrolled in an urban ART at two urban hospitals in Cameroon. Extended Cox regression procedures were used to identify significant predictors of retention in HIV care.

**Results:**

Of 455 patients, 314 (69%) were women, median (IQR) age and baseline CD4 cell count were respectively 36 years (30 – 43) and 110 cells/μL (39 – 177). Forty patients (9%) had active tuberculosis (TB) at enrollment. After a median (IQR) follow-up of 18 months (10–18), 346 (75%) were still in care, 8 (2%) were known dead, and 101 (22%) were lost to follow-up (LFU). Severe immunosuppression (CD4 cell count ≤ 50 cells/μL) at baseline (aHR 2.3; 95% CI 1.4 – 3.7) and active tuberculosis upon enrollment (aHR 1.8; 95% CI 1.0 – 3.6) were independent predictors of cohort losses to follow-up within the first 6 months after HAART initiation.

**Conclusion:**

These data suggest that three-quarter of HIV patients initiated on HAART remained in care and on HAART by 18 months; however, those with compromised immunologic status at treatment initiation, and those co-infected with TB were at increased risk for being lost to follow-up within the first 6 months on treatment.

## Background

With more than 60% of the world’s HIV-infected people, sub-Saharan Africa is severely hit by the HIV epidemic; in 2007 alone, 76% of the estimated 2.1 million AIDS related death occurred in the region.[1] The advent of Highly Active Antiretroviral Therapy (HAART) has substantially changed the clinical course and management of HIV/AIDS, leading to comparable virologic and immunologic outcomes among HIV infected patients in high and low-income countries.[2–5] Countries in sub-Sahara Africa are rapidly scaling up access to antiretroviral treatment (ART) as part of a multi-pronged strategy to mitigating the devastating impact of the HIV epidemic.[1] HIV disease as a chronic condition requires long term retention of patients in care, as first step to ensuring optimal clinical outcomes. [6, 7]

Proportions of non-death losses to follow-up (LFU) varying from 2 to 59% have been reported across ART programs in sub-Saharan Africa.[8–10] However, few programs in sub-Saharan Africa have described the patterns and ascertained determinants of retention in care among patients initiating HAART. Moreover, there were conflicting reports on the role of HIV patients’ immunological status on their retention in care.[11, 12] It remains unclear whether severely immuno-compromised patients and those whose health status improved on HAART are less likely to remain in care.

In this study, we describe retention in care in an urban pilot ART program in Cameroon, and identify temporal distribution and determinants of losses to follow-up.

## Methods

### Treatment and follow-up

From March 1, 2003 to June 30, 2004, HAART eligible patients aged 15 years and older, with limited financial resources as defined by a standardized indigence score scale, were initiated on HAART and followed during 18 months in a pilot Antiretroviral Therapy (PART) initiative. This initiative of the Ministry of Public Health was launched in March 2003 with support from the World Bank and aimed at improving access to highly subsidized HAART and laboratory tests to indigent patients. Clinicians assessed each patient’s clinical and immunological eligibility for HAART while social workers assessed patient’s ability to pay using an indigence score scale which defines the patient’s co-payment ( table 1 ). Laboratory tests were fully subsidized. At baseline, and every 6 months after HAART initiation, each patient received a laboratory test package, including CD4 cell count, full blood count, transaminases, amylase, and blood glucose level. CD4 cell count and plasma HIV-RNA were quantified at the Centre Pasteur du Cameroun (CPC), a reference laboratory in Yaoundé.

A physician prescribed first- or second-line HAART regimens according to the 2002 Cameroonian guidelines.[13] First-line regimens combined 2 nucleoside reverse transcriptase inhibitors (NRTI) with 1 non-nucleoside reverse transcriptase inhibitors (NNRTI). These “NNRTI-based” regimens included nevirapine (NVP) or efavirenz (EFV) in combination with stavudine (d4T)/lamivudine (3TC) or zidovudine (AZT)/lamivudine (3TC). In 2003, the only fixed-dose combination (FDC) first-line regimen available in-country as generic was d4T/3TC/NVP (Triomune ^®^ , Cipla, Mumbai, India). Second-line protease inhibitor based regimen “PI-based” combined indinavir (IND)/ritonavir (RIT) with 1 nucleoside reverse transcriptase inhibitor (NRTI) backbone (3TC plus either AZT or d4T). The monthly cost of HAART regimens ranged from 27$ to $51.

Cotrimoxazole was prescribed for all the patients enrolled. Patients with pulmonary TB and a CD4 cell count > 200 cells/μL were treated for TB and HAART initiation was deferred. For TB patients with a CD4 cell count between 50 and 200 cells/μL, HAART was initiated after the completion of two months of TB therapy. TB patients with a CD4 cell count < 50 cells/μL were started simultaneously on TB therapy and HAART. Regimen for TB consisted of two months of isoniazid, rifampin, ethambutol, and pyrazinamide daily, followed by four months of isoniazid and rifampin daily. TB patients received their TB regimen at a TB clinic affiliated to the ART center.

Adherence nurses counseled patients on the importance of adherence prior to HAART initiation and encouraged them to designate a close relative who was also counseled on treatment adherence. Each patient received monthly appointments for drug dispensation and every three months for clinical evaluation. During the period under review, home-visits were not performed for patient who missed a scheduled visit.

### Retention in care

The study outcome, loss to follow-up, was defined as any patient who missed all follow-up visits and could not be contacted by telephone for 2 consecutive months and who did not re-enter the study at a later date. Based on clinical records, we determined the length of follow-up for each patient as the elapsed time between HAART initiation and the last visit. Patients were regarded as being in care in a given month if they attended to follow-up visit within that month. For patients known to have died, the date and reported causes of death, when available, were recorded.

### Statistical analysis

Patients were included in the analysis regardless of any previous HAART at enrollment, and we excluded those with incomplete files (lacking key socio-demographic as age and gender, and immunologic information as baseline CD4 cell count). All patient information was censored at the 18 ^th^ month after enrollment or on the date of data collection (January 31, 2005). We computed rates and 95% confidence intervals using exact binomial procedure. To detect differences between groups, we used the Pearson Chi-square test statistic for categorical variables, and the 2-tailed t test statistic for continuous variables. In case of any violation of the assumption of equality of variance, we reported the unpaired t-test statistics; otherwise, the paired t-test was reported. We used the Kaplan-Meier method to estimate the cumulative probability of remaining in care. We plotted smoothed hazard-function estimators of losses to follow-up with the SAS macro %Smooth using a kernel smoothing method.[14] We defined as “early losses to follow-up” and “late losses to follow-up”, those occurring within and after the first 6 months following HAART initiation.

We first built a multivariate model to assess the probability of retention in care, considering the event of interest as any loss to follow-up and defining a censored observation for patient remaining in care, or known dead. In a second model, we assessed the ‘net probability’ of retention in care, considering death as a competing outcome. In this later worst-case scenario model, the event of interest was defined as any loss to follow-up or notified death.[15] We used the log-rank test to examine statistical difference between levels of the predictors: gender, age, baseline CD4 cell count, WHO clinical stage, patient co-payment for HAART, and active tuberculosis upon enrollment.

We retained baseline CD4 cell count, active tuberculosis upon enrollment as significant predictors (p value <0.25) for the Cox’s model building and controlled for treatment site and previous history of HAART. We assessed the proportional hazard assumption using the graphical method. Since the variables baseline CD4 cell count and active tuberculosis violated the proportional hazard assumption, we defined time-dependent variable based on the pattern of crude hazard function. Based on the pattern of the hazard function, we defined two time-intervals (≤ 6 months, and > 6 months). For each predictor, we defined two time-dependent variables as product-terms with each time-interval and we used an extended Cox’ proportional hazard model to derived adjusted hazard ratio.[15] Collinearity was assessed with the macro %Collin (SAS Institute, Inc, Cary, NC).[16] We used SAS version 9.1, SAS Institute Inc, Cary, NC, USA for these analyses. All p-values were two-sided and the significance level of 0.05 was used for all statistical tests.

## Results

### Baseline characteristics

**Figure 1:**
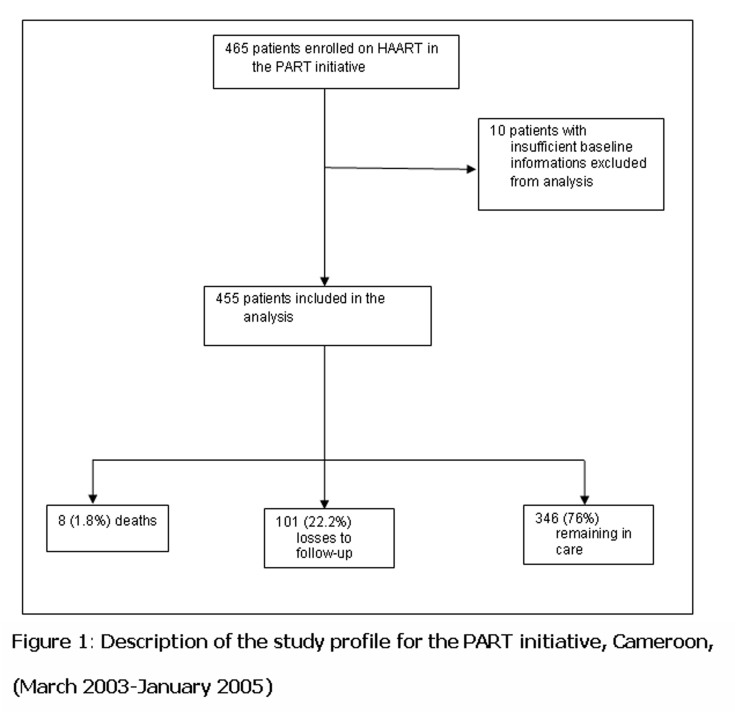
Description of the study profile for the PART initiative, Cameroon, (March 2003–January 2005)

Of the 465 individuals who started HAART between March 1, 2003 and June 30, 2004, 455 individuals (98%) were included in final analysis ( figure 1 ). Baseline characteristics of patients are summarized in table 2 . Of these, 314 (69%) were women; the median (IQR) age was 36 years (30–43). A total of 171 patients (38%) were enrolled at WHO stage III/IV. Forty (9%) patients were diagnosed with active tuberculosis at enrollment.

### Retention in Care

**Figure 2:**
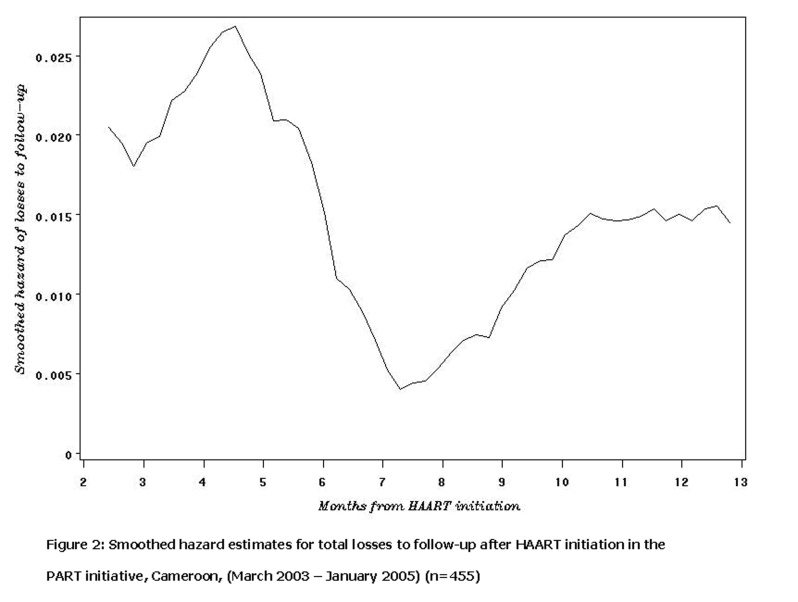
Smoothed hazard estimates for total losses to follow-up after HAART initiation in the PART initiative, Cameroon, (March 2003–January 2005) (n=455)

Of the 455 patients enrolled and followed-up during a median (IQR) period of 18 (10–18) months, 346 (76%) remained in care, 8 (1.8%) were reported dead and 101 (22.2%) were lost to follow-up ( figure 1 ). Patients lost to follow-up differed from those who remained in care by site, baseline CD4 cell count and the presence of active tuberculosis. Of note in table 2 , at baseline, there was a significant difference in terms of severe immunosuppression between patients remaining in care and those who subsequently dropped-out (29% vs. 44%; p=0.04). In addition, the latter were twice as likely to have TB/HIV co-infection at baseline (7% vs. 14%; p=0.04). The pattern of losses to follow-up varied with time ( figure 2 ), and depicted three distinct phases: an early high risk phase during the first 6 months (early losses to follow-up), during which almost two-third of the losses were registered; followed by a sharp decrease of the risk between 6 and 8 months, and a steady increase after 9 months. .

Early losses were associated with significantly lower mean CD4 cell count/μL at baseline compared to patients remaining in care (76 vs. 122; two sided t-test p value < 0.001). Conversely patients lost to follow-up after 6 months on HAART (late losses to follow-up) had comparable mean CD4 cell count/μL than those remaining in care, respectively at baseline (139 vs. 122; two sided t-test p =0.3) and 6 months (280 vs. 286; two sided t-test p =0.8), however, late losses to follow-up had significantly higher mean CD4 cell count/μl at 12 month as compared to those remaining in care (236 vs. 327; two sided t-test p =0.02).

**Figure 3:**
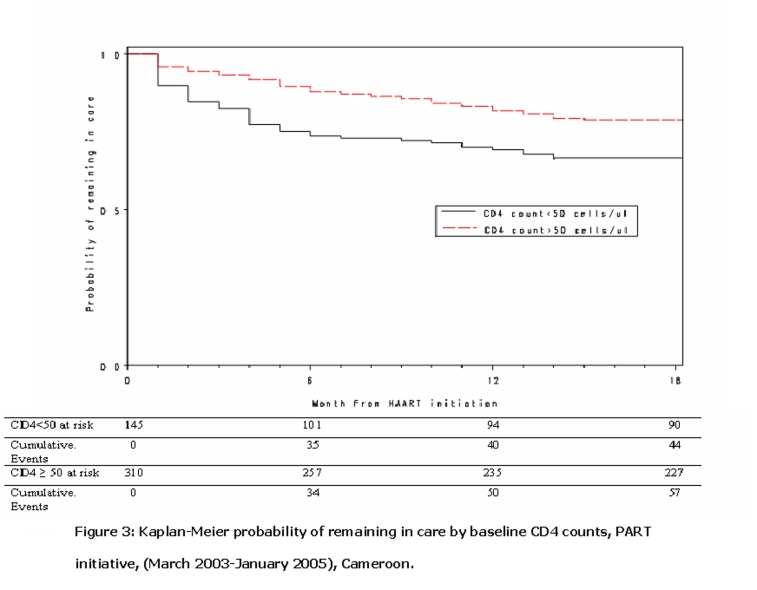
Kaplan-Meier probability of remaining in care by baseline CD4 counts, PART initiative, March 2003–January 2005), Cameroon.

**Figure 4:**
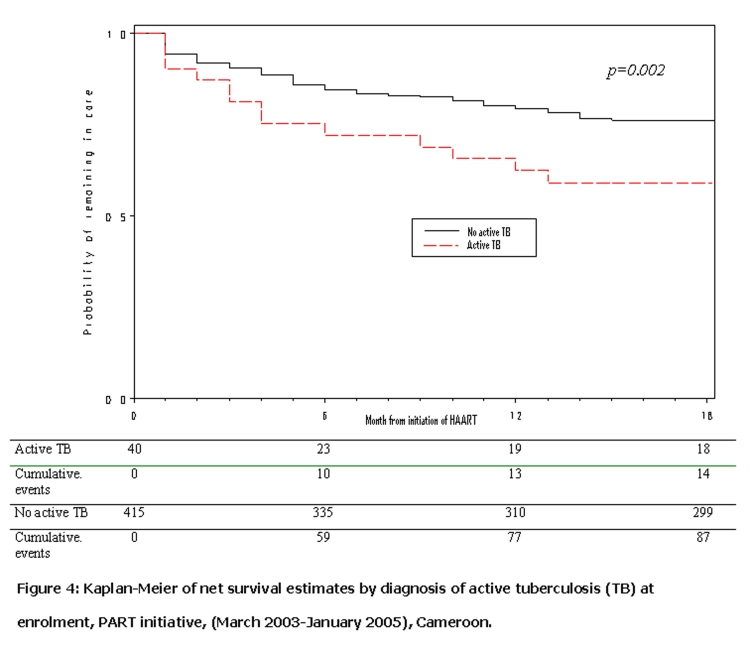
Kaplan-Meier of net survival estimates by diagnosis of active tuberculosis (TB) at enrolment, PART initiative, (March 2003–January 2005), Cameroon.

Kaplan-Meier curves ( figure 3 and figure 4 ) show that patients with baseline CD4 cell count > 50 cells/μL (p=0.002) and without active tuberculosis at enrollment (p=0.02) had increased probability of remaining in care In an extended Cox model patients with baseline CD4 cell count < 50 cells/μL were 2.3 times more likely than those with baseline CD4 cell count > 50 cells/μL to be lost from the program in the first 6 months after HAART initiation (adjusted hazard ration (aHR)= 2.3; 95% CI 1.4 – 3.7) ( table 3 ). Similarly, patients diagnosed with active TB at enrollment were 1.8 times more likely than those free of TB to be lost to follow-up in the first 6 months after HAART initiation (aHR= 1.8; 95% CI 1–3.6).

### “Net retention” in care

For the 455 patients followed during 537.5 person-year, the mortality rate was 1.5 per 100 person-years (95% CI 0.5 – 2.7). In a worst-case scenario, the probability of remaining in care and alive (net retention) was 0.83 (95% CI 0.79–0.86) at 6 month, 0.77 (95% CI 0.73–0.81) at 1 year, and 0.75 (95% CI 0.71–0.78) at 18 months. Patients with severe immuno suppression at baseline (CD4 cell count < 50 cells/μL) were 2.3 times more likely to be lost to follow-up by 6 months after HAART initiation (aHR= 2.3; 95% CI 1.5 – 3.7) ( table 3 ).

## Discussion

One-third of the patients in this program were enrolled at an advanced stage of HIV disease. After 18 months, 75% of patients remained alive and in care despite the lack of outreach activities and defaulters tracking system in the community. Unlike other reports from ART programs in low-income [8–10] and high-industrialized countries [11], we described a pattern of program drop-out among patients initiating HAART. In the first 6 months drop-out (early losses) occurred significantly among patients with severe immunosuppression or with active TB at enrollment. Likewise, we reported an increased risk of program drop-out among patients on HAART for 6 months or more (late losses). Late losses were not associated with severe immunosuppression at baseline; however, those patients showed similar CD4 cell count gain at 6 months and a significantly greater gain at 12 months compared to those remaining in care. This could imply therefore, a possible role of health status improvement on health care seeking behaviors in HIV patients on HAART. Patients could have resume activities which were incompatible with the frequency of hospital visits, or might have decided to transfer to another treatment center without notification. Moreover, the hidden cost of care in ART (transportation, nutrition, patient’s financial participation to ART treatment cost as defined by the indigence score, treatment of opportunistic infections, etc...) cannot be underestimated in a long term care model.

Nine percent of patients were diagnosed with TB upon enrollment. None of the reported deaths was attributed to TB, although, patients with active TB at enrollment were more likely to drop out from the program in the first 6 months after initiation of HAART as compared to those without TB. Patients diagnosed with TB upon enrollment in the PART initiative received TB treatment in a TB clinic located either within the same hospital where the ART clinic is found or away from the ART clinic. For instance, in the Douala Laquintinie Hospital, TB clinic and ART services coexisted in the same hospital, contrary to Yaoundé Central Hospital which had to refer TB patients to an external facility (Jamot Hospital in Yaoundé) located at a distance of 6 km away. However, these patients were still considered enrolled in the ART cohort at Yaoundé Central Hospital where they came for ARV drugs refill. This difference in location might have contributed to the high proportion of loss to follow-up observed in Yaoundé Central hospital compared to Douala Laquintinie hospital ( table 1 ).

Community support for ART programs, including tracing and referral of treatment defaulters has been reported to significantly improve ART programs outcomes in sub-Saharan Africa [17, 18]. Lower rates of losses to follow-up (6–12%) have been reported in ART Programs with active follow-up of patients on HAART over years [12, 17, 19] . We reported 23% of program losses over 18 months in the PART initiative without outreach activities. This proportion of losses would likely have been reduced if such strategy was implemented. However, some authors are questioning the cost-effectiveness of conducting outreach patient tracing; in a recent analysis from an ART program in Zambia, [17] an average of 19 home-visit attempts were necessary to generate a single return for care among patients lost to follow-up who were alive.

Our results suggest however, the need for more pro-active strategies to prevent early losses to follow-up, including identifying HIV patients early through routine provider initiated HIV counseling and testing (PITC), and strengthening integration of TB/HIV co-infection management.

This program evaluation was conducted on selected sites enrolled in an initiative to increase access to HIV care and treatment for indigent Patients, less likely to have accessed HAART otherwise at that time. This ART program was initiated at an early stage of ART scale-up in Cameroon, and additional strategies were subsequently integrated in 2006 to strengthen communities outreach activities (tracing system for treatment defaulters, home visits and home-based care).

## Conclusion

Despite the affordability of ART services, HIV patients enrolled with severe immunosuppression, and those co-infected with TB were more likely to be lost to follow-up early after HAART initiation. HIV programs should prioritize strategies for early identification and enrollment of HIV patients prior to advanced stage of disease, as well as strengthening the management of TB/HIV co-infected patients. Strategies for long term adherence counseling should be designed and implemented to ensure retention in care of patients with substantial immuno recovery. Further studies are needed to understand the determinants of late losses to follow-up.

## Figures and Tables

**Table 1:** Indigence score-scale used to define patient monthly HAART co-payment

Criteria	Scoring Scale
1- Number of children	No children = 0 1–2 = 1 3–5 = 2 more than 5 = 3
2- Number of other Dependents (adults and children)	No other dependents = 0 1 dependent = 1 2 or more dependents = 2
3- Number of dependents who is person living with HIV/AIDS(PLHA)	No = 0 One or more in care and not on HAART = 2 One or more on HAART = 3
4- Source of financial support	Insurance cooperative = 0 Personal income =1 Family support= 2 None = 3
5- Housing conditions	Own a home = 0 Rent a home = 1 Paid by third party = 2
6- Access to food	No difficulties to access food= 0 Some difficulties to access food = 1 Monthly difficulties to access food = 2 Daily difficulties to access food = 2
7- Monthly income	$0 – $19 = 5 $20 – $45. = 4 $46 – $91 = 3 $92 – $136 = 2 $137 – $182 = 1

** Total indigence score **	** Patient co-payment for HAART **

** > 16 **	0%
** 14–16 **	25%
** 10–13 **	50%
** 4–9 **	75%
** <4 **	100%

**Table 2:** Baseline characteristics of patients who either remained in the program, or were lost to follow-up in the PART initiative, Yaoundé and Douala, Cameroon, (March 2003– January 2005)

** Characteristic **	** All **	** Remained in the program a**	** Lost to follow-up **	***p***
** Demographic, (no. = 455) **		354 (77·8)	101 (22·2)	
Female	314 (69)	247 (70)	67 (66)	* NS *
Age, mean (SD), years	37 (9)	37·5 (9)	37 (9)	* NS *
Yaoundé	290 (63·7)	216 (61)	74 (73)	· * 02 *
** WHO stage at HAART initiation (no. = 455) **				
Stage I/II	274 (62)	217 (63)	57 (58)	* NS *
Stage III/IV	171 (38)	130 (37·5)	41 (42)	* NS *
** CD4 counts (no. = 455) **				
Mean (SD), cells per μL	110 (39 – 177)	121 (111 – 133)	96 (76 – 116)	· * 02 ***d**
Count < 50 cells per μL	145 (32)	101 (29)	44 (44)	· * 004 ***d**
** Active tuberculosis **	40 (9)	26 (7)	14 (14)	· * 04 ***d**
** HAART regimen b (no. = 455) **				
d4T/ 3TC/ NVP	135 (30)	116 (33)	19 (19)	* NS *
d4T/ 3TC/ EFV	103 (23)	74 (21)	29 (29)	* NS *
AZT/ 3TC/ IND	77 (17)	54 (15)	23 (23)	* NS *
AZT/ 3TC/ EFV	71 (16)	57 (16)	14 (14)	* NS *
d4T/ 3TC/ RIT	57 (12)	44 (12)	13 (13)	* NS *
Other **c**	12 (2)	9 (2·5)	3 (3)	* NS *
** HAART-naïve (no. = 452) **	395 (87·4)	308 (87·5)	87 (87)	* NS *
** Patient co-payment for HAART (%) e (no. = 432) **							
> 50	23 (5·3)	19 (5·7)	4 (4·1)	* 0.05 ***d**
49 – 25	187 (43·3)	134 (40·1)	53 (54·1)	
< 25	222 (51·4)	181 (54·2)	41 (41·8)	

**Note:** Data are no. (%) of patients, unless otherwise indicated. Statistical comparisons were made between characteristics of those who were observed (retained in care, or known death) compared with the characteristics of those who were lost to the program. SD, standard deviation.

a These include patients remaining alive in care and those known death.

b HAART (d4T: Stavudine; 3TC: Lamivudine; NVP: Nevirapine; AZT: Zidovudine; IND: Indinavir; RIT: Ritonavir; ddI: didanosine)

c AZT/ 3TC/ NVP; AZT/ ddI/ EFZ; IND/ddI/NFV; 3TC/ddI/ EFZ.

d P < .05 (We used Pearson Chi-square for categorical and 2-tailed t-test for continuous variables) NS, non significant.

e HAART monthly cost shared by patient defines the percentage of the monthly HAART cost that patients paid out-of-pocket, and this was calculated using a scoring scale described in table 1.

**Table 3:** Results of separate Cox’s models predicting relative hazard of early or late lost to follow-up and death after HAART initiation in the PART initiative, Yaoundé and Douala, Cameroon, (March 2003– January 2005)

	** Death **	** First 6 months **		** Death **	** After 6 months **	
		** Loss to follow-up **	** Death or lost to follow-up a**		** Loss to follow-up **	** Death or lost to follow-up a**
** Variable **						

** CD4 counts **						
≥50	1.0	1.0	1.0	1.0	1.0	1.0
< 50	1.8 (0.3–10.7)	2.3 (1.4–3.7) **b**	2.3 (1.5–3.7) **b**	1.4 (0.1–16.3)	1.0 (0.4–2.0)	1.0 (0.5–2.1)
** Active TB **						
No	1.0	1.0	1.0	1.0	1.0	1.0
Yes	....	1.8 (1–3.6) **b**	1.6 (0.8–3.2)	.....	2.2 (0.8–6.5)	1.9 (0.7–5.6)

Note. Data are adjusted hazard ratio (95% CI). Hazard ratios were estimated for the time-to-event outcomes of death, lost to follow-up and death or lost to follow-up using an extended Cox model allowing for a time-dependent variable (SAS PHREG Procedure). We defined for each variable in the model, a product term with a heavy side function of time (before and after 6 month) based on the pattern of the survival curves. Analyses were adjusted on site, WHO stage, previous HAART. TB, tuberculosis.

a We performed sensitivity analysis to estimate to hazard of death in a worst-case scenario where we considered all the losses to follow-up as deaths.

b Significant hazard ratio at 5% level of significance.
